# Police-reported family violence victimisation or perpetration and mental health-related emergency department presentations: an Australian data-linkage study

**DOI:** 10.1186/s12889-023-17570-y

**Published:** 2024-01-09

**Authors:** Nina Papalia, Melanie Simmons, Michael Trood, Troy McEwan, Benjamin Spivak

**Affiliations:** Centre for Forensic Behavioural Science, Swinburne University of Technology and Victorian Institute of Forensic Mental Health (Forensicare), Level 1/582 Heidelberg Road, Alphington, VIC 3078 Australia

**Keywords:** Family violence, Victimisation, Perpetration, Self-harm, Mental illness, Substance abuse, Data linkage, Longitudinal

## Abstract

**Background:**

Family violence is a leading social determinant of mental ill-health but its link to mental health-related emergency department presentations is poorly understood. Existing research has largely used retrospective designs with a focus on victimisation, typically among women. We examined whether police-reported family violence victimisation and perpetration were prospectively associated with mental health emergency department presentations in women and men. We also identified family violence risk and vulnerability characteristics associated with such presentations.

**Methods:**

Demographics, prior police involvement, and individual and relationship vulnerabilities were provided by Victoria Police for 1520 affected family members (i.e., primary victims) and 1470 respondents (i.e., persons alleged to have perpetrated family violence) from family violence reports in 2016–17. Emergency mental health presentations 22–30 months post-family-violence report were determined through linkage with the Victorian Emergency Minimum Dataset and compared to statewide presentations.

**Results:**

Emergency mental health presentations during follow-up were identified in 14.3% of the family violence sample, with 1.9% presenting for self-harm. Mental health presentation rates per 1,000 people were markedly higher among affected family members and respondents of both sexes and all ages than in the general population, except for male affected family members aged 45 + . Adjusting for age and sex, the mental health presentation rate was 6 and 11 times higher among affected family members and respondents, respectively, than in the general population. Individual vulnerabilities were more closely related to risk of emergency mental health presentations than relationship characteristics.

**Conclusions:**

Police-recorded family violence is associated with increased mental health-related emergency department presentations over the short-to-medium term. Strengthened cross-sector collaboration is needed to identify, address, and refer individuals with overlapping family violence and mental health needs and to improve victims’ and perpetrators’ access to community mental health and related services. This should help prevent individuals from reaching a crisis point in their mental health.

**Supplementary Information:**

The online version contains supplementary material available at 10.1186/s12889-023-17570-y.

## Background

In 2020–21, there were 309,657 emergency department (ED) presentations for mental-health-related reasons across Australia [1]. Several states have noted a concerning trend of increasing mental health ED presentations over time, not explained by population growth [1, 2], with similar trends observed internationally [3, 4]. People experiencing a mental health crisis may receive care in EDs for a variety of concerns, including self-harm and suicidality, psychosis, alcohol and/or drug-related problems, mood disturbances, and acute stress. Research suggests that individuals presenting to EDs for mental health-related reasons are at increased risk of repeat presentations [5] and adverse outcomes such as suicide [6, 7] and other deaths [8]. Given the highly distressing and costly nature of mental health ED presentations and current pressures on the Australian public healthcare system, it is critical we develop a better understanding of these presentations, and their risk factors, to identify opportunities to prevent crises and reduce the growing burden on EDs.

### Relationship between family violence and mental health ED presentations

Family violence is a leading social determinant of mental ill-health in Australia and internationally [9–13], however its link to emergency mental health presentations is under-researched. Two Australian retrospective studies highlight an association between self-reported family violence and mental health ED presentations. First, research from the Northern Territory suggested that almost half of individuals who attended the ED for mental health reasons reported exposure to family violence or conflict [14]. Research in New South Wales found that almost two-thirds of women who attended the ED in suicidal crises had a history of intimate partner abuse victimisation, with a third of victimised women reporting abuse within 18 months before presentation [15]. These findings are consistent with international research [16, 17].

In the relatively small body of literature linking family violence and mental health ED presentations, most studies focus on victimisation, typically among women. The relationship between family violence perpetration and mental health ED presentations is poorly understood. This is a curious omission given that perpetrators who disclose suicidal ideation require immediate action due to the potential link to lethal behaviour [18]. Moreover, MacIsaac and colleagues’ [19] Victorian study of the relationship between family violence and suicide found that perpetration appeared more temporally related to suicide than victimisation. Notably, 35% of males identified as perpetrators had engaged in family violence within six weeks of their death by suicide, whereas victimisation for both males and females appeared to be more distally related to the suicide (i.e., family violence victimisation  > 12 months earlier).

### Adopting a ‘whole-of-government approach’ to reducing mental health ED presentations

The Royal Commission into Victoria’s Mental Health System [20] highlighted the need for a ‘whole-of-government approach’ to improving the mental health and wellbeing of Victorians, rather than separating the mental health system from the systems that address the social determinants of mental ill-health. A number of services, such as police, social welfare, and family violence services, interact with individuals who experience or engage in family violence and may be able to contribute to a whole-of-government approach by providing early identification of individuals at increased risk of crisis mental health presentations and linking them into supports. Further, Victoria’s Royal Commission into Family Violence [21] highlighted a need for mental health services to better assess and identify individuals who are experiencing or engaging in family violence, noting that despite being one of the leading contributors to mental-ill health, mental health services struggle to identify and support those affected by both family violence and mental illness.

To help inform a whole-of-government approach to improving mental health outcomes, it would be useful to ascertain what factors increase the risk of mental health ED presentations among those who have had police involvement for family violence. Although not all family violence is reported to police, once reported, police collect a wide range of data on all parties involved in a family violence incident. Leveraging this data and knowledge may be beneficial to identifying individuals at risk of mental health ED presentations. For instance, it may be that some of the individual (e.g., mental health concerns, substance use) or relationship characteristics (e.g., recent separation, financial difficulties, pregnancy or new birth, escalation in behaviour) related to future family violence [22] may trigger or exacerbate a mental health crisis and subsequent emergency care. Indeed, some of these factors (mental health concerns, substance use) have already been identified as related to future ED presentations in non-family-violence cohorts [23, 24]. If these factors are identified by police (or by family violence-sector workers and related services), at-risk individuals may be offered pre-crisis support to reduce the likelihood of later mental health ED presentations.

### Aims of the current study

The prospective relationship between police-reported family violence (victimisation or perpetration) and mental health-related ED presentations has not been examined in detail. Without this knowledge, it is unclear whether police or healthcare services are adequately meeting the needs of this population or can do more to prevent mental health crises that may lead to ED presentations, which are distressing and costly. To help address these gaps, this study aimed to: 1) compare the rate of mental health ED presentations in 2,900 individuals involved in police-reported incidents of family violence (through victimisation or perpetration) in Victoria, Australia, with that of the general population; and 2) examine whether family violence-related risk and vulnerability factors identified by police at the time of family violence report were associated with risk of future mental health ED presentations.

## Methods

### Design

Data were drawn from a pseudo-prospective data-linkage study evaluating the effect of a novel policing strategy on family violence recidivism and health outcomes. The intervention was implemented in two metropolitan Victoria Police divisions between 2016 and 2018. A routine-practice control sample of police-reported family violence incidents was drawn from a neighbouring division during the same period and is the sample for this analysis. Administrative police records were obtained for all individuals and probabilistically linked by an independent linkage agency to administrative health datasets. For this study, we used health records from the Victorian Emergency Minimum Dataset (VEMD).

### Data sources

#### Family violence sample and risk assessment data

The sample was extracted from Victoria Police’s Law Enforcement Assistance Program (LEAP), an electronic database recording all contacts between police and the public in Victoria, including family violence incidents. Victoria Police are directed by the Family Violence Protection Act 2008 [25] to identify an incident as family violence when it involves any family member (e.g., partner, child, parent, etc.) engaging in physical (e.g., assault, sexual assault, etc.) or non-physical (e.g., coercion, threats, psychological abuse, etc.) behaviour that causes another family member fear for their safety or the safety of another. Police record all family violence incidents to which they respond, irrespective of whether the behaviour constitutes a criminal offence, with around half of incidents leading to charges [26]. Police always identify an alleged^1^ perpetrator (respondent) and primary victim (affected family member) in family violence records.

The current sample comprised 2,990 unique individuals, including 1470 respondents and 1520 affected family members. These individuals were from a sample of 1,590 LEAP-recorded family violence incidents involving unique dyads that were reported to Victoria Police North-Western Division 4 between 1 July 2016 and 28 February 2017^2^; these incidents were randomly selected from all incidents reported to this police division over this period (*N* = 3,942 incidents). From the 1,590 dyads, seven dyads were removed due to missing identifying information, and the remaining dyads were split into 3,166 individuals. For individuals who appeared multiple times across dyads (*n* = 162), we selected their earliest family violence incident reported during the sampling frame, leaving the final sample of 2,990 unique individuals.

The following LEAP information was extracted for everyone: demographics; evidence-based family violence risk and vulnerability factors relating to the incident, the affected family member, the respondent, and their relationship recorded by police at the time of the ‘index’ family violence report (i.e., the incident report leading to inclusion in the sample); and prior police involvement for family violence. Variables used in this study included: sex; age at index report; recorded role in index incident (affected family member or respondent); relationship between affected family member and respondent at index incident (intimate partner, child/parent, or other); prior LEAP-recorded family violence report as respondent (yes/no) or affected family member (yes/no); and individual and relationship vulnerabilities recorded by police at the index family violence report (see Table 2 in Appendix).

#### Family violence sample ED presentations

ED presentations were obtained from VEMD, which contains person-level records of all presentations to Victorian public hospitals with designated EDs. It records demographic (e.g., sex, age), presentation (e.g., arrival date, length of stay), and clinical (e.g., injury cause, diagnosis) data for each presentation. Linkage of the family violence sample to VEMD was conducted by the Centre for Victorian Data Linkage utilising probabilistic linkage with Victoria Police-provided identifiers (name, sex, birth date). Linkage and VEMD data extraction were undertaken in 2021, with VEMD coverage from 1 July 2000 through 31 December 2018.

Two VEMD variables were used to determine mental health presentations: (1) primary diagnosis, established to be mainly responsible for ED attendance and provided in International Classification of Diseases Revision 10 Australian Modification (ICD-10-AM) format; and (2) human intent, reflecting a clinician’s assessment of the most likely human intent in any injury. Mental health presentations were defined as those leading to an ICD-10-AM F-code diagnosis (F00-F99; *mental illness* presentations) or those with a diagnosis of physical injury (T/S code) combined with human intent coded as intentional self-harm (*self-harm* presentations) [2]. A final set of diagnostic codes associated with mental health presentations (e.g., accidental drug poisoning, laceration of wrist with undetermined intent) [27] were grouped as *other mental health* presentations (see Table 3 in Appendix). These three subtypes of mental health presentations were combined to form an *any mental health* presentations category.

‘Future’ mental health presentations in the family violence sample were defined in two ways to address the two aims. For the first aim, a count of mental health presentations was calculated by summing the incidence of each individual’s presentations for 22 months from 28 February 2017 (end date of family violence sampling period) to 31 December 2018. We selected the end date of the sampling period to ensure that everyone in the family violence sample had been exposed to family violence. For the second aim (and for computing descriptive statistics), a binary indicator was constructed to measure any mental health presentations between the index family violence report (ranged from 1 July 2016 and 28 February 2017) to 31 December 2018, providing follow-up of 22–30 months. We also created a variable reflecting any history of mental health ED presentations from July 2000 to the index family violence report.

#### General population ED presentations

Statewide population-level mental health ED presentations were obtained from the Victorian Agency for Health Information (VAHI). We obtained all mental health ED presentations recorded on VEMD from 28 February 2017 to 31 December 2018, disaggregated by sex, age at presentation (≤ 17, 18–24, 25–34, 35–44, and 45 +), and type of presentation, using the same definitions of mental health ED presentations as the family violence sample. VAHI does not hold person-level records; therefore, analyses comparing the family violence sample and general population were at the aggregate/presentation level.

### Ethics approval

Ethical approval with a participant consent waiver was granted by the Australian Institute of Health and Welfare Ethics Committee (EO2017/5/380) and Swinburne University Human Research Ethics Committee (ID-572). Permission to access police and health records was granted by Victoria Police and the Department of Health, respectively. All methods were carried out in accordance with the relevant guidelines and regulations.

### Analyses

To address the first research aim, ED presentation counts from 28 February 2017 to 31 December 2018 were calculated by age group, sex, and sample (affected family members; respondents; general population), for any mental health presentations and the three subtypes (mental illness, self-harm, and other mental health). Population rates for mental health ED presentations were analysed descriptively by calculating rates per 1,000 people across age groups and sex in each sample; to conserve space, we provide a figure depicting rates for any mental health presentations, and figures for the three subsidiary presentation types are provided in the Appendix. Given that individuals aged at least one year during the follow-up period, age groups for the family violence affected family members and respondents were calculated using each person’s age at the midway point of the follow-up period. Rates were calculated by dividing the presentation count by the size of the sample and multiplying by 1,000. Estimates of the general population size by age and sex were obtained from the 2016 Australian Census using location on census night [28].

Differences in mental health ED presentation rates between the family violence sample and the general population were then investigated using models adjusting for age and sex, with role in the index family violence incident (respondent or affected family member) included as a term. Separate models were constructed for any mental health presentations and the three subsidiary types. One of three types of generalised linear models (Poisson, Quasi-Poisson, or Negative Binomial) was used. Where both the Poisson and Negative Binomial models’ standard errors were overdispersed, the Quasi-Poisson model was used. Where two or more of the three models did not evidence overdispersion, the model with the lowest Akaike Information Criterion (AIC) or quasi-AIC was used [29]. The natural logarithm of each group’s sample or population size was used as an offset in each model. The model coefficients, incident rate ratios (IRRs) and their 95% confidence intervals, were exponentiated for ease of interpretation.

To address the second research aim, multivariate logistic regression models were constructed predicting the probability of a future mental health ED presentation (i.e., between the index family violence report and 31 December 2018) from family violence risk and vulnerability factors, separately for affected family members and respondents. For these analyses, we only examined any mental health ED presentations as the outcome (not the three subsidiary types) to maximise the number of observations and reduce complexity. When constructing the models, thematic blocks of variables were added to each subsequent model to assess the extent to which adjusting for each set of variables influenced the association between the initial set and outcome variable. Restricted cubic splines were fitted to age to address non-linearity, with three-to-five-knot spline specifications compared on the AIC; differences were negligible (≤ 2) so three knots were chosen. Model coefficients are presented as odds ratios (ORs) with 95% confidence intervals (CIs). Nagelkerke *R*^2^ is presented for each model as measure of model fit.

## Results

### Descriptive characteristics

Table 1 reports the characteristics of the family violence sample and the prevalence of mental health ED presentations, disaggregated by subtype (any, mental illness, self-harm, and other) and, for mental illness ED presentations, by common primary diagnosis subtypes. Individuals with multiple mental health ED presentation subtypes (and/or multiple mental illness ED presentations with different primary diagnosis subtypes) during the follow-up period are counted within each subcategory but contribute once to the aggregate category; therefore, subtypes of presentations do not sum to the relevant total.

**Table 1:** Descriptive characteristics and future mental health emergency department presentations (from the index family violence report to December 31 2018) in the family violence sample

	Total Sample *N* = 2,990	AFMs *n* = 1520	Respondents *n* = 1470	*p* value
	*n* (%)	*n* (%)	*n* (%)	
Sex^a^				
Male	1,555 (52.1%)	410 (27.0%)	1,145 (77.9%)	
Female	1,431 (47.9%)	1,107 (73.0%)	324 (22.1%)	< 0.001
Age at index FV report^b^, *M* (*SD*)	36.10 (14.48)	36.48 (16.16)	35.71 (12.51)	0.15
≤ 17 years	222 (7.5%)	152 (10.2%)	70 (4.8%)	
18–24 years	432 (14.7%)	211 (14.1%)	221 (15.3%)	
25–34 years	784 (26.7%)	369 (24.7%)	415 (28.6%)	
35–44 years	728 (24.8%)	330 (22.1%)	398 (27.5%)	
45 + years	775 (26.4%)	430 (28.8%)	345 (23.8%)	< 0.001
Relationship type at index FV report^c^				
Intimate partner	1,755 (58.7%)	881 (58.0%)	874 (59.5%)	
Child/parent^d^	720 (24.1%)	375 (24.7%)	345 (23.5%)	
Other family	513 (17.2%)	263 (17.3%)	250 (17.0%)	0.68
Any prior (pre-index FV report) police-reported FV	1,932 (64.6%)	912 (60.0%)	1,020 (69.4%)	< 0.001
Prior police-reported FV as respondent	1,365 (45.7%)	458 (30.1%)	907 (61.7%)	< 0.001
Prior police-reported FV as AFM	1,333 (44.6%)	805 (53.0%)	528 (35.9%)	< 0.001
Any prior (pre-index FV report) mental health ED presentation	795 (26.6%)	365 (24.0%)	430 (29.3%)	0.001
Any future (post-index FV report) mental health ED presentation	427 (14.3%)	175 (11.5%)	252 (17.1%)	< 0.001
Mental illness ED presentation (ICD-10 code)	275 (9.2%)	107 (7.0%)	168 (11.4%)	< 0.001
Substance use-related (F10-F19)	125 (4.2%)	40 (2.6%)	85 (5.8%)	< 0.001
Schizophrenia, schizotypal & delusional (F20-F29)	83 (2.8%)	25 (1.6%)	58 (3.9%)	< 0.001
Mood (F30-F39)	60 (2.0%)	23 (1.5%)	37 (2.5%)	0.068
Stress- and anxiety-related (F40-F48)	55 (1.8%)	22 (1.4%)	33 (2.2%)	0.14
Adult personality disorders (F60-F69)	18 (0.6%)	8 (0.5%)	10 (0.7%)	0.76
Child behavioural/emotional (F90-F98)	16 (0.5%)	7 (0.5%)	9 (0.6%)	0.75
Self-harm ED presentation	58 (1.9%)	20 (1.3%)	38 (2.6%)	0.017
Other mental health ED presentation	258 (8.6%)	106 (7.0%)	152 (10.3%)	0.001

*FV* Family violence, *ED* Emergency department, *AFM* Affected family member (i.e., primary victim), Respondent, person alleged to have perpetrated family violence. Where data are missing, percentages are expressed as a proportion of the valid (non-missing) *n* rather than column total *n*

^a^4 cases (3 affected family members, 1 respondent) had missing sex. Victoria Police record only binary sex categories, meaning these two categories likely include a small number of non-binary identifying individuals

^b^49 cases (28 affected family members, 21 respondents) had missing age

^c^2 cases (1 affected family member, 1 respondent) had missing relationship type

^d^This relationship type is non-directional and could involve parental abuse or child abuse

Overall, there were 1188 post-index-family-violence report mental health ED presentations in the sample, 932 (78.5%) of which were triaged as level 1–3 (potentially to immediately life-threatening). There were 427 (14.3%) individuals with at least one future mental health presentation (any), including 275 individuals (9.2%) with a mental illness presentation, 58 individuals (1.9%) with a self-harm presentation, and 258 individuals (8.6%) with other mental health presentations (Table 1). Respondents (i.e., persons alleged to have perpetrated family violence) were significantly more likely than affected family members (i.e., primary victims) to have a future mental health ED presentation (17.1% versus 11.5%, respectively). Where individuals had a mental illness presentation, the most prevalent primary diagnoses recorded as mainly responsible for ED attendance were psychoactive substance use-related, schizophrenia, schizotypal and delusional disorders, and mood disorders. Among individuals with any future mental health ED presentation, the mean number of presentations during follow-up was 2.78 (*SD* = 3.70, range: 1–32), with no significant differences between respondents (*M* = 2.90, *SD* = 3.59, range: 1–27) and affected family members (*M* = 2.61, *SD* = 3.85, range: 1–32), *t*(425) = -0.82, *p* = 0.41.

### Association between family violence and mental health ED presentations (Aim 1)

**Fig. 1:**
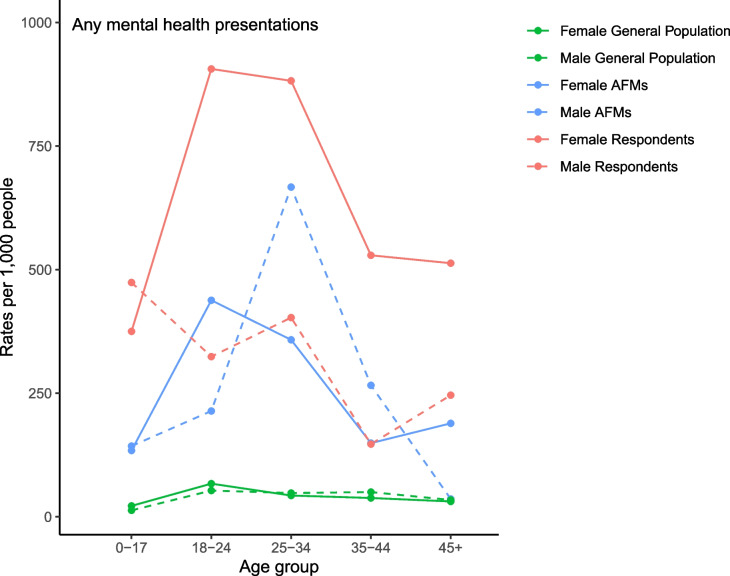
Mental health emergency department presentation (any) rates per 1,000 people (from 28 February 2017 to 31 December 2018) for family violence affected family members, respondents, and the general population, by age group and sex. *Legend.* AFM, affected family member (i.e., primary victim); Respondent, person alleged to have perpetrated family violence. The data for this graph are included in the Appendix, Table A4

Figure 1 shows that the mental health (any) ED presentation rates per 1,000 people were higher among family violence affected family members and respondents of both sexes and all age groups relative to the general population, except for male affected family members aged 45 + where no obvious difference was observed. Rates were generally highest for females identified as respondents, except for those aged ≤ 17, where rates were highest among male respondents. The same overall patterns emerged for mental illness presentations (see Figure A1 in Appendix). Similar trends were observed for self-harm and other mental health presentations – female respondents generally had the highest rates, except for those aged 18–24, where male respondents had the highest self-harm rates, and those aged 25–34, where male affected family members had the highest rates of other mental health presentations (see Figures A2 and A3 in Appendix). Caution must be taken when interpreting rate estimates in the family violence sample, which were sometimes based on very small population denominators (e.g., 16 female and 38 male respondents aged ≤ 17 years; see Table A4 in Appendix).

Table 2 reports the IRRs from models examining age- and sex-adjusted differences in mental health ED presentations for family violence affected family members and respondents compared with the general population. Respondents and affected family members had significantly higher presentation rates than the general population, except for rates of self-harm presentations among affected family members, where increases were not statistically significant. IRRs were larger for respondents than affected family members, though confidence intervals were overlapping.

**Table 2:** Age- and sex-adjusted mental health emergency department presentations (from 28 February 2017 to 31 December 2018) among family violence affected family members and respondents compared with the general population: incident rate ratios and 95% confidence intervals

Variable	Any mental health ED presentations^NB^	Mental illness ED presentations^NB^	Self-harm ED presentations^QP^	Other mental health ED presentations^QP^
	IRR (95% CI), *p* value	IRR (95% CI), *p* value	IRR (95% CI), *p* value	IRR (95% CI),* p* value
Intercept	0.03 (0.02,0.04), < .001	0.01 (0.01,0.01), < .001	< .01 (< .01, < .01), < .001	0.01 (0.01,0.01), < .001
Role in index FV incident				
FV AFMs	6.01 (4.27,8.45), < .001	6.78 (1.57,18.19), < .001	3.80 (0.24,16.32), 0.19	5.90 (1.51,15.10), < .001
FV respondents	11.45 (8.17,16.04), < .001	8.80 (2.70,20.61), < .001	8.28 (1.17,27.30), 0.01	8.36 (2.76,18.83), < .001
Male	0.79 (0.60,1.05), 0.1	1.10 (0.99,1.22), 0.09	0.57 (0.49,0.65), < .001	0.98 (0.90,1.07), 0.7
Age				
18–24	2.33 (1.45,3.72), < .001	4.22 (3.42,5.24), < .001	3.08 (2.50,3.79), < .001	2.82 (2.36,3.38), < .001
25–34	2.54 (1.59,4.08), < .001	3.39 (2.77,4.18), < .001	1.64 (1.33,2.04), < .001	2.21 (1.86,2.63), < .001
35–44	1.51 (0.94,2.42), 0.08	3.32 (2.70,4.11), < .001	1.24 (0.98,1.58), 0.09	2.12 (1.78,2.54), < .001
45 +	1.19 (0.74,1.90), 0.47	1.98 (1.63,2.41), < .001	0.61 (0.49,0.76), < .001	2.18 (1.88,2.53), < .001

*ED* Emergency department, *AFM* Affected family member (i.e., primary victim), Respondent, person alleged to have perpetrated family violence, *IRR* Incident rate ratio, *CI* Confidence interval, *NB* Negative binomial model, *QP* Quasi-poisson model, *FV* Family violence, *FV* AFMs/respondents reference = general population sample, Male reference = Female, Age reference = 0–17 years of age

### Risk and vulnerability factors for mental health ED presentations in the family violence sample (Aim 2)

Tables 3 and 4 present the adjusted ORs for associations between family violence risk and vulnerability factors and future mental health ED presentations (any), among affected family members and respondents, respectively (see Tables A5 and A6 in Appendix for descriptives and unadjusted associations). For affected family members, police-identified depression or mental health issues, suicidal ideation/attempts, and drug use were associated with significantly increased odds of a mental health ED presentation in Model 1. These associations were robust to the inclusion of relationship vulnerabilities (Model 2), where the latter variables were not significantly related to mental health ED presentations. After adding historical violence and prior mental health ED presentations (Model 3), depression or mental health issues, suicidal ideation/attempts, and drug use remained moderately-to-strongly associated with mental health ED presentations; though prior police-reported family violence perpetration and prior mental health ED presentations were also significant predictors. These relationships remained significant and of similar magnitude in Model 4 (adjusting for sex and age), in which prior mental health ED presentations demonstrated the strongest association with future mental health presentations.

**Table 3:** Adjusted ORs and 95% CIs from logistic regression models predicting affected family members’ future mental health ED presentations (any; from the index family violence report to 31 December 2018) from family violence risk and vulnerability factors recorded by police as present at the index incident report

Risk or Vulnerability Factor	Model 1, *N* = 1520	Model 2,* N* = 1520	Model 3, *N* = 1520	Model 4, *N* = 1490
**AFM Individual Factors**				
Depression or mental health issues	2.36 [1.52, 3.69]***	2.40 [1.53, 3.77]***	1.69 [1.04, 2.75]*	1.74 [1.07, 2.84]*
Suicidal ideation or attempts	3.62 [1.43, 9.18]**	3.78 [1.48, 9.64]**	3.53 [1.30, 9.60]*	3.11 [1.15, 8.43]*
Isolation	0.58 [0.29, 1.13]	0.50 [0.25, 1.02]	0.51 [0.24, 1.09]	0.50 [0.23, 1.06]
Alcohol use	1.02 [0.63, 1.66]	1.00 [0.61, 1.63]	0.91 [0.54, 1.54]	1.00 [0.59, 1.69]
Drug use	3.85 [2.48, 5.98]***	3.97 [2.53, 6.22]***	2.38 [1.46, 3.87]***	2.19 [1.34, 3.57]**
**Relationship Factors**				
Recent separation	–	1.08 [0.65, 1.77]	1.10 [0.65, 1.84]	1.09 [0.64, 1.85]
Escalation	–	1.05 [0.66, 1.68]	1.22 [0.74, 2.01]	1.32 [0.80, 2.20]
Financial difficulties	–	1.00 [0.56, 1.76]	1.06 [0.58, 1.94]	1.15 [0.62, 2.12]
Harm or threat to harm the AFM	–	0.75 [0.46, 1.21]	0.74 [0.44, 1.23]	0.72 [0.43, 1.21]
Sexual assault of AFM	–	0.69 [0.22, 2.15]	0.56 [0.17, 1.92]	0.36 [0.10, 1.26]
Controlling behaviours by respondent	–	1.51 [0.97, 2.36]	1.41 [0.87, 2.29]	1.43 [0.88, 2.33]
Pregnancy or recent birth	–	0.95 [0.42, 2.16]	0.90 [0.38, 2.15]	0.78 [0.32, 1.90]
**Hx of Mental Health and Violence**				
Prior mental health ED presentation	–	–	5.45 [3.76, 7.89]***	5.26 [3.60, 7.66]***
Prior police reported FV as AFM	–	–	0.82 [0.56, 1.21]	0.92 [0.61, 1.39]
Prior police reported FV as respondent	–	–	1.97 [1.35, 2.87]***	2.04 [1.35, 3.07]***
**Nagelkerke R**^**2**^	0.10	0.11	0.24	0.26

*FV* Family violence, *ED* Emergency department, *AFM* Affected family member (i.e., primary victim), Respondent, person alleged to have perpetrated family violence, *Hx* History, *OR* Odds ratio, *CI* Confidence interval. Model 1 contains only AFM individual variables, Model 2 contains AFM and relationship variables; Model 3 adjusted for all variables in Model 2 as well as adjustments for prior mental health ED presentations and police-reported family violence, Model 4 contains the same variables as Model 3 as well as adjustments for sex and age at index incident (30 cases deleted due to missingness)

^*^p < 0.05, **p < 0.01, ***p < 0.001

**Table 4:** Adjusted ORs and 95% CIs from logistic regression models predicting respondents’ future mental health ED presentations (any; from the index family violence report to December 31 2018) from family violence risk and vulnerability factors recorded by police as present at the index incident report

Risk or Vulnerability Factor	Model 1, *N* = 1470	Model 2, *N* = 1470	Model 3, *N* = 1470	Model 4, *N* = 1449
**Respondent Individual Factors**				
Depression or mental health issues	2.21 [1.57, 3.10]***	2.37 [1.67, 3.35]***	1.67 [1.14, 2.43]**	1.65 [1.13, 2.43]*
Suicidal ideation or attempts	2.78 [1.53, 5.06]***	3.08 [1.66, 5.71]***	3.48 [1.76, 6.88]***	3.40 [1.71, 6.77]***
Unemployment	1.02 [0.66, 1.55]	1.15 [0.74, 1.78]	1.08 [0.68, 1.74]	1.08 [0.67, 1.74]
Alcohol use	1.63 [1.18, 2.24]**	1.69 [1.22, 2.33]**	1.67 [1.17, 2.37]**	1.84 [1.29, 2.64]***
Drug use	1.64 [1.19, 2.25]**	1.70 [1.23, 2.35]**	1.33 [0.93, 1.89]	1.27 [0.88, 1.83]
**Relationship Factors**				
Recent separation	–	0.61 [0.36, 1.03]	0.71 [0.41, 1.24]	0.73 [0.42, 1.27]
Escalation	–	1.07 [0.72, 1.60]	1.10 [0.71, 1.71]	1.07 [0.68, 1.66]
Financial difficulties	–	0.57 [0.31, 1.02]	0.60 [0.33, 1.10]	0.60 [0.32, 1.11]
Harm or threat to harm the AFM	–	1.00 [0.67, 1.48]	0.95 [0.61, 1.49]	0.99 [0.63, 1.55]
Sexual assault of AFM	–	0.68 [0.22, 2.11]	0.62 [0.18, 2.17]	0.54 [0.15, 1.89]
Controlling behaviours by respondent	–	0.54 [0.34, 0.84]**	0.55 [0.34, 0.90]*	0.57 [0.35, 0.94]*
Pregnancy or recent birth	–	0.86 [0.40, 1.84]	1.20 [0.53, 2.75]	1.28 [0.56, 2.91]
**Hx of Mental Health and Violence**				
Prior mental health ED presentation	–	–	6.93 [5.00, 9.59]***	6.66 [4.79, 9.26]***
Hx of violent behaviour by respondent	–	–	0.82 [0.49, 1.38]	0.86 [0.51, 1.45]
Prior police reported FV as AFM	–	–	1.25 [0.90, 1.72]	1.22 [0.86, 1.72]
Prior police reported FV as respondent	–	–	0.92 [0.64, 1.32]	1.05 [0.72, 1.52]
**Nagelkerke R**^**2**^	0.09	0.12	0.28	0.30

*FV* Family violence, *ED* Emergency department, *AFM* Affected family member (i.e., primary victim), Respondent, person alleged to have perpetrated family violence, *Hx* History, *OR* Odds ratio, *CI* Confidence interval. Model 1 contains only respondent individual variables, Model 2 contains respondent and relationship variables, Model 3 adjusted for all variables in Model 2 as well as adjustments for prior mental health ED presentations, general violence, and police-reported family violence, Model 4 contains the same variables as Model 3 as well as adjustments for sex and age at index incident (21 cases deleted due to missingness)

^*^p < 0.05, **p < 0.01, ***p < 0.001

For respondents, police-identified depression or mental health issues, suicidal ideation/attempts, drug use, and alcohol use were associated with increased odds of a mental health ED presentation (Model 1). These associations remained significant when relationship-level factors were included (Model 2), though controlling behaviour by the respondent toward the affected family member resulted in reduced odds of a respondent having a mental health ED presentation. Prior mental health ED presentations were strongly associated with increased odds of a future mental health ED presentation, whereas prior police-reported family violence victimisation or perpetration were non-significant (Model 3). In the fully adjusted model (Model 4), suicide ideation/attempts and a history of mental health ED presentations remained strongly associated with mental health ED presentations, while depression or mental health issues, alcohol use, and controlling behaviour were also significant predictors.

## Discussion

More than 1 in 4 individuals in contact with police for family violence had a mental health ED presentation before their index family violence report, while 1 in 7 had a mental health presentation in the approximately two-year follow-up period. Compared with the general population, mental health ED presentation rates per 1,000 people were markedly higher among family violence affected family members (i.e., primary victims) and respondents (i.e., persons alleged to have perpetrated abuse) of both sexes and all age groups, except male affected family members aged 45 + . Adjusting for age and sex, the rate of mental health presentations was 6 and 11 times higher among affected family members and respondents, respectively, than the general population. Several police-identified family violence risk and vulnerability factors were associated with mental health ED presentations.

The findings support prior retrospective research showing disproportionately high rates of family violence victimisation in individuals presenting to EDs in crisis [14, 15]. We advance this literature by demonstrating a prospective association between police-recorded family violence and mental health ED presentations, which extends to those identified as perpetrating family violence. Respondents had about eight times the rate of mental illness-related, self-harm, and other mental health ED presentations than the general population, and female respondents generally had the highest presentation rates per 1,000 people. This may suggest that women with family violence behaviours have higher rates of mental illness and substance use than men with similar behaviours. This is broadly consistent with findings from the general violence literature showing that females with severe mental illness have a higher relative risk of violent offending compared to males, despite only accounting for a very small portion of all violent crimes [30]. This interpretation should be considered in conjunction with information about the relationship in which family violence is occurring. Partner violence was the most common relationship type at the index family violence incident among both male (63.6%) and female respondents (45.1%). However, female respondents were more often identified as perpetrating violence in child/parent relationships at the index incident (37%) relative to male respondents (19.7%) (noting that this relationship category is non-directional and could involve abuse of a parent or abuse of a child). It is possible therefore that the higher rate of mental health ED presentations found among female respondents could reflect differences in the mental health needs of individuals who are violent in different kinds of family relationships. These matters are nuanced and require greater exploration in future work where sample size allows for disaggregation of relationship types. A third potential reason for this result is that women who experience mental illness or use substances may be more likely to be misidentified by police as respondents, when they are primarily victim-survivors [31]. It was not possible to determine the presence of potential misidentification based on the data available to us. Regardless of the reasons for these results, they add to a very limited literature in Australia on the mental health needs of individuals identified as perpetrating family violence [32], and suggest that this is an area in need of further investigation for both men and women.

The findings suggest that data routinely collected by police to inform family violence risk management may also have utility in identifying those at greater risk of emergency mental health presentations. Depression or mental health issues, suicidal ideation/attempts, and substance use concerns (i.e., affected family members’ drug use and respondents’ alcohol use) flagged at the index report were robust predictors of mental health ED presentations for affected family members and respondents, consistent with research in non-family violence samples [23, 24]. Further work is needed to understand whether family violence influences these relationships. It may be that the connections between individual mental health vulnerabilities and crisis ED presentations are exacerbated (or partly mediated) by family violence. Alternatively, in the lead up to mental health crises, individuals may engage in increasingly erratic behaviour in the home environment which could attract police attention due to concerned family members or neighbours, particularly when young people are involved.

Relationship characteristics did not significantly predict mental health ED presentations in multivariable models, except for respondent controlling behaviours, which were linked to reduced odds of respondents presenting in crisis. This requires further investigation but may indicate less acute mental health needs/distress and/or help-seeking among those known to use controlling behaviours. Some research suggests that those who use controlling behaviours are more likely to be ‘specialists’ (i.e., perpetrators of family violence alone) rather than ‘generalists’ (i.e., perpetrators of family violence and other offending), and generalists have more significant mental health needs and substance use problems [33–35].

Regarding the role of prior police-recorded family violence, only past perpetration predicted future mental health presentations in the affected family member sample, in fully adjusted models. This is broadly consistent with Australian and international research showing that the co-occurrence of victimisation and offending is associated with higher rates of mental ill-health [36–38]. The lack of significant effect for historical family violence victimisation in affected family member and respondent models does not necessarily mean that prior victimisation does not influence risk of crisis presentations to EDs. A more plausible interpretation may be that any influence likely operates indirectly through more proximal factors (e.g., mental health issues, suicidal ideation, substance use concerns), which themselves may be consequences of past victimisation [39, 40]. Further longitudinal research is needed to examine these temporal pathways. Despite the relevance of police-identified family violence risk and vulnerability factors to crisis ED presentations, having a prior mental health ED presentation was the strongest predictor of a future mental health ED presentation in fully adjusted models for respondents and affected family members, in line with other reports [5, 24].

### Limitations

Our comparisons to the general population provide a baseline for understanding the associations between family violence and mental health ED presentations, rather than indicating a causal nexus. Although we adjusted for age and sex, we could not account for other confounders that might have influenced both involvement in police-recorded family violence and later mental health crisis presentations to EDs (e.g., socioeconomic status, social isolation), potentially leading to inflated IRR estimates; future work should endeavour to account for such confounding influences. Conversely, the use of a general population sample rather than a non-family violence-involved control group likely had a downward influence on IRR estimates. General population ED presentations were only available at the presentation level, meaning to address Aim 1, we needed to select a timeframe for comparison across which aggregate presentation counts could be generated for the family violence sample. Although the selected timeframe (i.e., end date of the family violence sampling period to 31 December 2018) ensured everyone in the family violence sample had been exposed to family violence, it meant that any ED presentations that occurred earlier in the sampling period (among those sampled earlier) were not counted in analyses related to Aim 1.

Ascertaining family violence (and associated vulnerability factors) through administrative police records is highly conservative as most family violence is unreported. Additionally, our analyses aggregated family violence cases occurring within intimate and nonintimate family relationships. While we consider this a strength given the known interrelationships between, and common causes of, different forms of relational violence [41], we acknowledge that there are also likely to be differences in the dynamics and drivers of abuse occurring in different family relationships. Future work could therefore explore whether the links between family violence and mental health ED presentations vary by family relationship type.

The VEMD does not include private ED presentations and underrepresents regional/rural presentations that do not meet VEMD reporting requirements [42]. As participants were sampled from a metropolitan police division, this may reduce the likelihood of missed regional/rural presentations. Although our approach to coding ‘other’ mental health ED presentations was informed by prior work [27], it is possible some presentations (e.g., accidental drug poisoning) were not mental health-related. That said, physical health presentations related to underlying mental health concerns were likely missed due to VEMD’s one-diagnosis-per-presentation format.

Despite these limitations, our study had several strengths. We used a large, contemporary sample that is broadly representative of all family violence cases reported to the relevant police division. The data linkage methodology allowed the sample to be tracked continuously without the attrition that can threaten longitudinal survey studies, and allowed us to determine the temporal ordering of events without the potential for inaccuracy or bias due to self-reports of sensitive information (e.g., family violence, mental health). Although VEMD diagnostic codes are not independently validated, their integrity is regularly reviewed by an external advisory group [2].

### Implications and conclusions

Results from this study indicate that individuals with police involvement for family violence – whether for victimisation or perpetration, and including males, females, children, and adults – have elevated rates of emergency mental health presentations compared to the general population. Clinicians and services working with individuals, couples, or families in the context of family violence should be cognisant of this association and consider what supports may be needed to prevent crises that may result in emergency care, particularly for clients with known mental health concerns, substance use, and past suicidal ideation/attempts. Relatedly, staff working in EDs and other acute mental health settings may benefit from increased training to enquire about family violence, given it has likely been experienced or perpetrated relatively recently by a significant portion of individuals in their care [14, 15] and such knowledge may help identify those at increased risk of subsequent crisis presentations to EDs. Australian research suggests that screening practices for family violence perpetration (as opposed to victimisation) in mental health service settings are limited and inconsistent [43]. This is potentially a missed opportunity to identify and respond to family violence and understand and address its possible impact on mental health crises (and vice versa).

Alongside enhanced mental health supports for victims, findings underscore the importance of improving perpetrators’ access to community mental health and related services to reduce the need for acute ED care and to advance suicide prevention efforts [19, 44]. As 1 in 7 family violence-involved individuals had a mental health ED presentation in the approximately two-year follow-up period, police may be well placed to identify and respond to individuals at increased risk. This requires police–mental health sector collaboration on continued training, along with clear referral pathways to community mental health and drug and alcohol services, especially where concerns in these areas are identified at the time of family violence report. Similarly, strengthening how EDs and other crisis services link in with community mental health/drug and alcohol services, family support services (particularly for young people), and specialist family violence services may improve continuity of care and help reduce further crises leading to emergency care (and so ease the growing burden on EDs). Further longitudinal research is needed to understand possible causal and bidirectional pathways between an individual experiencing or engaging in family violence and them reaching a crisis point in their mental health that may result in ED attendance. This may help identify early warning signs and targets for intervention, ultimately improving individual and community safety.

### Supplementary Information

**Additional file 1: Table A1. **Characteristics of the general population covered by the police division in which family violence incident reports were sampled in the study (Victoria Police North-West Division 4, or ND4), compared with the characteristics of the Victorian general population. **Table A2.** Individual and relationship risk and vulnerability factors from LEAP family violence index incident reports. **Table A3.** Emergency department presentation codes included as mental health presentations. **Table A4.** Raw data used to compute mental health presentation rates per 1,000 people (from 28 February 2017 to 31 December 2018) by age group and sex for family violence AFMs, respondents, and general population. **Figure A1.** Mental illness emergency department presentation rates per 1,000 people (from 28 February 2017 to 31 December 2018) for family violence affected family members, respondents, and the general population, by age group and sex. *Legend*. AFM, affected family member (i.e., primary victim); Respondent, person alleged to have perpetrated family violence. The data for this graph are included in Table A4. **Figure A2.** Self-harm emergency department presentation rates per 1,000 people (from 28 February 2017 to 31 December 2018) for family violence affected family members, respondents, and the general population, by age group and sex. *Legend.* AFM, affected family member (i.e., primary victim); Respondent, person alleged to have perpetrated family violence. The data for this graph are included in Table A4. **Figure A3.** Other mental health emergency department presentation rates per 1,000 people (from 28 February 2017 to 31 December 2018) for family violence affected family members, respondents, and the general population, by age group and sex. *Legend*. AFM, affected family member (i.e., primary victim); Respondent, person alleged to have perpetrated family violence. The data for this graph are included in Table A4. **Table A5.** Unadjusted ORs and 95% CIs representing the associations between family violence risk and vulnerability factors recorded by police at the index incident report and future mental health ED presentations (any; from the index family violence report to December 31 2018), among family violence affected family members. **Table A6.** Unadjusted ORs and 95% CIs representing the associations between family violence risk and vulnerability factors recorded by police at the index incident report and future mental health ED presentations (any; from the index family violence report to December 31 2018), among family violence respondents.

## Data Availability

The datasets generated and/or analysed during the current study are not publicly available because our strict legal agreements with the data custodians do not allow us to make these sensitive health and personal data available to third parties. If anyone wishes to access the data on a reasonable request, they will need to first obtain the necessary permissions and approvals from the data custodians and ethics committees, which includes signing confidentiality deeds. Such permission/approval would be required from the Victorian Department of Health, Victoria Police, and the Australian Institute of Health and Welfare. The corresponding author can be contacted for information about this process.

## Abbreviations

ED: Emergency department
VEMD: Victorian Emergency Minimum Dataset
LEAP: Law Enforcement Assistance Program
ICD-10-AM: International Classification of Diseases Revision 10 Australian Modification
VAHI: Victorian Agency for Health Information
AIC: Akaike Information Criterion
IRR: Incident Rate Ratio
OR: Odds Ratio
CI: Confidence Interval
FV: Family Violence
AFM: Affected Family Member

## Footnotes

1 The term ‘alleged’ is implied in all subsequent references to family violence respondents and affected family members in the sample to recognise that: (a) many incidents do not result in a formal criminal charge; and (b) police identification of the ‘respondent’ and ‘affected family member’ may be influenced by an array of factors and relates to a given incident without looking at broader patterns of behaviour.

2 Population sociodemographic characteristics of the community covered by this western metropolitan division are presented in the Appendix (Table 1). The population is comparable to the statewide population on sex, education level, and median income; it includes more younger people (64.5% < 45 years vs 60.2%), fewer Indigenous Australians (0.57% vs 0.80%), and fewer individuals born in Australia (59.8% vs 64.1%), compared with the Victorian population.
