# Supplementary material for: Police-reported family violence victimisation or perpetration and mental health-related emergency department presentations: an Australian data-linkage study
Source: BMC Public Health. 2024 Jan 9;24:131. doi: 10.1186/s12889-023-17570-y (PMC10777561; doi:10.1186/s12889-023-17570-y)
Supplement: Supplementary file 1 — Additional file 1: Table A1. Characteristics of the general population covered by the police division in which family violence incident reports were sampled in the study (Victoria Police North-West Division 4, or ND4), compared with the characteristics of the Victorian general population. Table A2. Individual and relationship risk and vulnerability factors from LEAP family violence index incident reports. Table A3. Emergency department presentation codes included as mental health presentations. Table A4. Raw data used to compute mental health presentation rates per 1,000 people (from 28 February 2017 to 31 December 2018) by age group and sex for family violence AFMs, respondents, and general population. Figure A1. Mental illness emergency department presentation rates per 1,000 people (from 28 February 2017 to 31 December 2018) for family violence affected family members, respondents, and the general population, by age group and sex. Legend. AFM, affected family member (i.e., primary victim); Respondent, person alleged to have perpetrated family violence. The data for this graph are included in Table A4. Figure A2. Self-harm emergency department presentation rates per 1,000 people (from 28 February 2017 to 31 December 2018) for family violence affected family members, respondents, and the general population, by age group and sex. Legend. AFM, affected family member (i.e., primary victim); Respondent, person alleged to have perpetrated family violence. The data for this graph are included in Table A4. Figure A3. Other mental health emergency department presentation rates per 1,000 people (from 28 February 2017 to 31 December 2018) for family violence affected family members, respondents, and the general population, by age group and sex. Legend. AFM, affected family member (i.e., primary victim); Respondent, person alleged to have perpetrated family violence. The data for this graph are included in Table A4. Table A5. Unadjusted ORs and 95% CIs representing the associ [file 12889_2023_17570_MOESM1_ESM.docx]

**Appendix**

**Table A1.** Characteristics of the general population covered by the police division in which family violence incident reports were sampled in the study (Victoria Police North-West Division 4, or ND4), compared with the characteristics of the Victorian general population.

| Characteristic | ND4 Area Population | Victorian Population |
| --- | --- | --- |
|  | *N* = 476,993 | *N* = 5,946,059 |
| Number of family violence incident reports per 100,000 people^a^ | 1,184 | 1,233 |
| Males | 49.1% | 49.0% |
| Females | 50.9% | 51.0% |
| Age |  |  |
| 0-17 years | 22.2% | 21.8% |
| 18-24 years | 9.9% | 9.7% |
| 25-34 years | 17.9% | 15.1% |
| 35-44 years | 14.5% | 13.6% |
| 45+ years | 35.5% | 39.8% |
| Aboriginal and/or Torres Strait Islander people | 0.57% | 0.80% |
| Born in Australia | 59.8% | 64.1% |
| Education |  |  |
| Graduate level or higher | 20.5% | 19.7% |
| Highest level = level 3/4 certificate, advanced diploma, diploma, or school grade 10 or above | 41.2% | 43.2% |
| Highest level = school grade 9 or below | 8.2% | 7.1% |
| Median personal income bracket per annum^b^ | $33,800-$41,599 | $33,800-41,599 |

*Note.* The demographic figures and underlying *N*’s reported in the table were calculated from the 2016 Australian Census (Australian Bureau of Statistics. [2016] 2016 Census - Counting Persons, Place of Enumeration (MB)[Table Builder]. Accessed 14/10/2023). Family violence incident reports were conversely derived from Victorian Police data for the financial year 2017/18, as the same information for 2016 is not publicly available (Crime Statistics Agency. [2022]. Victoria Police Data Tables (2021-22). Accessed 14/10/2023, <https://files.crimestatistics.vic.gov.au/2023-04/Victoria%20Police%20Data%20Tables%20%282021-22%29.xlsx>). The underlying *N*’s to compute a rate of family violence incident reports per 100,000 people for ND4 and Victoria were therefore taken from the 2017 estimated resident population (Australian Bureau of Statistics. [2023]. ABS Estimated resident population 2001-2021 by 2021 LGA [BETA]. Accessed 20/10/2023, <https://digital.atlas.gov.au/datasets/019e1fed4e9f429eaf9189a0a461ead5_1/about>).

^a^ The underlying *N* for ND4 and Victoria were based on estimated resident population size as of 2017 to match the time period of family violence incident reports during this period: Victoria *N* = 6,299,798; ND4 *N* = 528,283;

^b^ The underlying *N* for ND4 and Victoria are based only on those individuals who reported an income in the 2016 census: Victoria *N* = 3,887,629, ND4 *N* = 312,571.

**Table A2.** Individual and relationship risk and vulnerability factors from LEAP family violence index incident reports.

| Risk or vulnerability factor | Definition |
| --- | --- |
| Depression or mental health issues | Scored by police indicating whether the AFM/respondent experienced depression or other mental health issues (y/n). This item is scored separately for AFMs and respondents. |
| Suicidal ideation or attempts | Scored by police indicating whether the AFM/respondent has expressed suicidal ideation or made a suicide attempt (y/n). This item is scored separately for AFMs and respondents. |
| Isolation | Scored by police indicating whether the AFM experienced isolation from their support network (y/n). |
| Unemployment | Scored by police indicating whether the respondent had problems with employment (y/n). |
| Alcohol use | Combination of two variables scored by police indicating whether the AFM/respondent probably or definitely had problems related to alcohol use (y/n). This item is scored separately for AFMs and respondents. |
| Drug use | Combination of two variables scored by police indicating whether the AFM/respondent probably or definitely had problems related to drug use (y/n). This item is scored separately for AFMs and respondents. |
| Recent separation | Scored by police indicating whether the AFM/respondent had recently separated (y/n). |
| Escalation | Scored by police indicating whether the police perceived that there had been an escalation (increase in severity and/or frequency) in abusive behaviour between the respondent and AFM (y/n). |
| Financial difficulties | Scored by police indicating whether the AFM/respondent experienced financial difficulties around the time of the family violence report (y/n). |
| Harm or threat to harm the AFM | Scored by police indicating whether the respondent harmed or threatened to harm the AFM (y/n). |
| Sexual assault of AFM | Scored by police indicating whether the respondent had sexually assaulted the AFM (y/n). |
| Controlling behaviours by respondent | Scored by police indicating whether the respondent had engaged in controlling behaviours toward the AFM (y/n). |
| Pregnancy or recent birth | Scored by police indicating whether the AFM/respondent was pregnant or had a recent birth (y/n). |
| History of violent behaviour by respondent | Scored by police indicating whether the respondent had any history of violent behaviour (y/n). |

*Note.* LEAP, Law Enforcement Assistance Program; AFM, affected family member (i.e., primary victim); Respondent, person alleged to have perpetrated family violence. Police score items based on their direct questioning and discretion (e.g., observing a party to be substance affected at the time of a report). Formal diagnoses were not required for the presence of a mental health or drug/alcohol use to be scored in the affirmative. Items recorded as absent (‘n’) may reflect true absence or missing data due to the “tick if present” format of the LEAP family violence incident reports. This means that the items operate on an “if the risk factor is known to be present” basis.

**Table A3.** Emergency department presentation codes included as mental health presentations.

| Variable | Description |
| --- | --- |
| Mental Illness Presentations | Any presentation with a primary diagnosis ICD-10-AM code of F00-F99. Subtypes of F-code diagnoses examined in descriptive analyses included:   - F10-F19: Psychoactive substance use-related - F20-F29: Schizophrenia, schizotypal and delusional disorders - F30-F39: Mood disorders - F40-F48: Stress- and anxiety-related - F60-F69: Adult personality disorders - F90-F98: Child behavioural and emotional disorders |
| Self-Harm Presentations | Any presentation with a primary diagnosis ICD-10-AM code of T or S (Injury, Poisoning & Certain Other Consequences of External Causes) AND where the VEMD ‘human intent’ variable is coded as = intentional self-harm |
| Other Mental Health Presentations | Any presentation with a primary diagnosis ICD-10-AM code of:   - R40-R46 – Symptoms and signs involving cognition, perception, emotional state and behaviour - S51 – open wound to forearm AND human intent variable is coded as = cannot be determined - S61 – open wound to wrist/hand AND human intent variable is coded as = cannot be determined - T18 – foreign body in alimentary tract (i.e., ingest foreign object) AND human intent variable is coded as = cannot be determined - T39 – Poisoning by non-opioid analgesics, antipyretics and anti-rheumatics AND human intent variable is coded as = accidental or intent cannot be determined - T40 – Poisoning by narcotics and psychodysleptics AND human intent variable is coded as = accidental or intent cannot be determined - T42 – Poisoning by antiepileptic, sedative-hypnotic and antiparkinsonism drugs AND human intent variable is coded as = accidental or intent cannot be determined - T43 – Poisoning by psychotropic drugs, not elsewhere classified AND human intent variable is coded as = accidental or intent cannot be determined - T51 – Toxic effect of alcohol AND human intent variable is coded as = accidental or intent cannot be determined - Z55-Z65 – Persons with potential health hazards related to socioeconomic and psychosocial circumstances - Z86.4 – Personal history of psychoactive substance abuse - Z86.5 – Personal history of other mental and behavioural disorders - Z91.5 – Personal history of self-harm |
| Any Mental Health Presentations | Any *Mental Illness* presentation, *Self-Harm* presentation, or *Other Mental Health* presentation |

*Note.* ICD-10-AM, International Classification of Diseases Revision 10 Australian Modification; VEMD, Victorian Emergency Minimum Dataset.

**Table A4.** Raw data used to compute mental health presentation rates per 1,000 people (from 28 February 2017 to 31 December 2018) by age group and sex for family violence AFMs, respondents, and general population.

| Age group | Sex | Sample | Population | Outcome | Presentations | Rates |
| --- | --- | --- | --- | --- | --- | --- |
| 0-17 | Female | AFMs | 82 | Mental Illness | np | np |
| 0-17 | Female | AFMs | 82 | Intentional Self-Harm | np | np |
| 0-17 | Female | AFMs | 82 | Other Mental Health | 7 | 85 |
| 0-17 | Female | AFMs | 82 | Any Mental Health | 11 | 134 |
| 18-24 | Female | AFMs | 153 | Mental Illness | np | np |
| 18-24 | Female | AFMs | 153 | Intentional Self-Harm | np | np |
| 18-24 | Female | AFMs | 153 | Other Mental Health | 36 | 235 |
| 18-24 | Female | AFMs | 153 | Any Mental Health | 67 | 438 |
| 25-34 | Female | AFMs | 285 | Mental Illness | 69 | 242 |
| 25-34 | Female | AFMs | 285 | Intentional Self-Harm | 10 | 35 |
| 25-34 | Female | AFMs | 285 | Other Mental Health | 23 | 81 |
| 25-34 | Female | AFMs | 285 | Any Mental Health | 102 | 358 |
| 35-44 | Female | AFMs | 249 | Mental Illness | 21 | 84 |
| 35-44 | Female | AFMs | 249 | Intentional Self-Harm | np | np |
| 35-44 | Female | AFMs | 249 | Other Mental Health | np | np |
| 35-44 | Female | AFMs | 249 | Any Mental Health | 37 | 149 |
| 45+ | Female | AFMs | 322 | Mental Illness | 30 | 93 |
| 45+ | Female | AFMs | 322 | Intentional Self-Harm | np | np |
| 45+ | Female | AFMs | 322 | Other Mental Health | np | np |
| 45+ | Female | AFMs | 322 | Any Mental Health | 61 | 189 |
| 0-17 | Male | AFMs | 56 | Mental Illness | np | np |
| 0-17 | Male | AFMs | 56 | Intentional Self-Harm | np | np |
| 0-17 | Male | AFMs | 56 | Other Mental Health | np | np |
| 0-17 | Male | AFMs | 56 | Any Mental Health | 8 | 143 |
| 18-24 | Male | AFMs | 42 | Mental Illness | 7 | 167 |
| 18-24 | Male | AFMs | 42 | Intentional Self-Harm | np | np |
| 18-24 | Male | AFMs | 42 | Other Mental Health | np | np |
| 18-24 | Male | AFMs | 42 | Any Mental Health | 9 | 214 |
| 25-34 | Male | AFMs | 69 | Mental Illness | 23 | 333 |
| 25-34 | Male | AFMs | 69 | Intentional Self-Harm | np | np |
| 25-34 | Male | AFMs | 69 | Other Mental Health | np | np |
| 25-34 | Male | AFMs | 69 | Any Mental Health | 46 | 667 |
| 35-44 | Male | AFMs | 94 | Mental Illness | 17 | 181 |
| 35-44 | Male | AFMs | 94 | Intentional Self-Harm | np | np |
| 35-44 | Male | AFMs | 94 | Other Mental Health | np | np |
| 35-44 | Male | AFMs | 94 | Any Mental Health | 25 | 266 |
| 45+ | Male | AFMs | 138 | Mental Illness | np | np |
| 45+ | Male | AFMs | 138 | Intentional Self-Harm | np | np |
| 45+ | Male | AFMs | 138 | Other Mental Health | np | np |
| 45+ | Male | AFMs | 138 | Any Mental Health | np | np |
| 0-17 | Female | Respondents | 16 | Mental Illness | np | np |
| 0-17 | Female | Respondents | 16 | Intentional Self-Harm | np | np |
| 0-17 | Female | Respondents | 16 | Other Mental Health | np | np |
| 0-17 | Female | Respondents | 16 | Any Mental Health | 6 | 375 |
| 18-24 | Female | Respondents | 53 | Mental Illness | np | np |
| 18-24 | Female | Respondents | 53 | Intentional Self-Harm | np | np |
| 18-24 | Female | Respondents | 53 | Other Mental Health | np | np |
| 18-24 | Female | Respondents | 53 | Any Mental Health | 48 | 906 |
| 25-34 | Female | Respondents | 85 | Mental Illness | 42 | 494 |
| 25-34 | Female | Respondents | 85 | Intentional Self-Harm | 8 | 94 |
| 25-34 | Female | Respondents | 85 | Other Mental Health | 25 | 294 |
| 25-34 | Female | Respondents | 85 | Any Mental Health | 75 | 882 |
| 35-44 | Female | Respondents | 87 | Mental Illness | 28 | 322 |
| 35-44 | Female | Respondents | 87 | Intentional Self-Harm | np | np |
| 35-44 | Female | Respondents | 87 | Other Mental Health | np | np |
| 35-44 | Female | Respondents | 87 | Any Mental Health | 46 | 529 |
| 45+ | Female | Respondents | 78 | Mental Illness | 18 | 231 |
| 45+ | Female | Respondents | 78 | Intentional Self-Harm | 6 | 77 |
| 45+ | Female | Respondents | 78 | Other Mental Health | 16 | 205 |
| 45+ | Female | Respondents | 78 | Any Mental Health | 40 | 513 |
| 0-17 | Male | Respondents | 38 | Mental Illness | 12 | 316 |
| 0-17 | Male | Respondents | 38 | Intentional Self-Harm | np | np |
| 0-17 | Male | Respondents | 38 | Other Mental Health | np | np |
| 0-17 | Male | Respondents | 38 | Any Mental Health | 18 | 474 |
| 18-24 | Male | Respondents | 148 | Mental Illness | 25 | 169 |
| 18-24 | Male | Respondents | 148 | Intentional Self-Harm | 6 | 41 |
| 18-24 | Male | Respondents | 148 | Other Mental Health | 17 | 115 |
| 18-24 | Male | Respondents | 148 | Any Mental Health | 48 | 324 |
| 25-34 | Male | Respondents | 320 | Mental Illness | 73 | 228 |
| 25-34 | Male | Respondents | 320 | Intentional Self-Harm | 8 | 25 |
| 25-34 | Male | Respondents | 320 | Other Mental Health | 48 | 150 |
| 25-34 | Male | Respondents | 320 | Any Mental Health | 129 | 403 |
| 35-44 | Male | Respondents | 327 | Mental Illness | 25 | 76 |
| 35-44 | Male | Respondents | 327 | Intentional Self-Harm | np | np |
| 35-44 | Male | Respondents | 327 | Other Mental Health | np | np |
| 35-44 | Male | Respondents | 327 | Any Mental Health | 48 | 147 |
| 45+ | Male | Respondents | 297 | Mental Illness | 45 | 152 |
| 45+ | Male | Respondents | 297 | Intentional Self-Harm | np | np |
| 45+ | Male | Respondents | 297 | Other Mental Health | np | np |
| 45+ | Male | Respondents | 297 | Any Mental Health | 73 | 246 |
| 0-17 | Female | General pop. | 630896 | Intentional Self-Harm | 2269 | 4 |
| 18-24 | Female | General pop. | 284974 | Intentional Self-Harm | 2898 | 10 |
| 25-34 | Female | General pop. | 457936 | Intentional Self-Harm | 2169 | 5 |
| 35-44 | Female | General pop. | 412672 | Intentional Self-Harm | 1358 | 3 |
| 45+ | Female | General pop. | 1248728 | Intentional Self-Harm | 2093 | 2 |
| 0-17 | Male | General pop. | 662729 | Intentional Self-Harm | 799 | 1 |
| 18-24 | Male | General pop. | 292022 | Intentional Self-Harm | 1337 | 5 |
| 25-34 | Male | General pop. | 441494 | Intentional Self-Harm | 1373 | 3 |
| 35-44 | Male | General pop. | 397126 | Intentional Self-Harm | 1068 | 3 |
| 45+ | Male | General pop. | 1117474 | Intentional Self-Harm | 1420 | 1 |
| 0-17 | Female | General pop. | 630896 | Mental Illness | 5808 | 9 |
| 18-24 | Female | General pop. | 284974 | Mental Illness | 9437 | 33 |
| 25-34 | Female | General pop. | 457936 | Mental Illness | 10217 | 22 |
| 35-44 | Female | General pop. | 412672 | Mental Illness | 8393 | 20 |
| 45+ | Female | General pop. | 1248728 | Mental Illness | 16755 | 13 |
| 0-17 | Male | General pop. | 662729 | Mental Illness | 3716 | 6 |
| 18-24 | Male | General pop. | 292022 | Mental Illness | 8507 | 29 |
| 25-34 | Male | General pop. | 441494 | Mental Illness | 12146 | 28 |
| 35-44 | Male | General pop. | 397126 | Mental Illness | 11455 | 29 |
| 45+ | Male | General pop. | 1117474 | Mental Illness | 17599 | 16 |
| 0-17 | Female | General pop. | 630896 | Other Mental Health | 5997 | 10 |
| 18-24 | Female | General pop. | 284974 | Other Mental Health | 6808 | 24 |
| 25-34 | Female | General pop. | 457936 | Other Mental Health | 7485 | 16 |
| 35-44 | Female | General pop. | 412672 | Other Mental Health | 6010 | 15 |
| 45+ | Female | General pop. | 1248728 | Other Mental Health | 20245 | 16 |
| 0-17 | Male | General pop. | 662729 | Other Mental Health | 3880 | 6 |
| 18-24 | Male | General pop. | 292022 | Other Mental Health | 5623 | 19 |
| 25-34 | Male | General pop. | 441494 | Other Mental Health | 7686 | 17 |
| 35-44 | Male | General pop. | 397126 | Other Mental Health | 7149 | 18 |
| 45+ | Male | General pop. | 1117474 | Other Mental Health | 19154 | 17 |
| 0-17 | Female | General pop. | 630896 | Any Mental Health | 14074 | 22 |
| 0-17 | Male | General pop. | 662729 | Any Mental Health | 8395 | 13 |
| 18-24 | Female | General pop. | 284974 | Any Mental Health | 19143 | 67 |
| 18-24 | Male | General pop. | 292022 | Any Mental Health | 15467 | 53 |
| 25-34 | Female | General pop. | 457936 | Any Mental Health | 19871 | 43 |
| 25-34 | Male | General pop. | 441494 | Any Mental Health | 21205 | 48 |
| 35-44 | Female | General pop. | 412672 | Any Mental Health | 15761 | 38 |
| 35-44 | Male | General pop. | 397126 | Any Mental Health | 19672 | 50 |
| 45+ | Female | General pop. | 1248728 | Any Mental Health | 39093 | 31 |
| 45+ | Male | General pop. | 1117474 | Any Mental Health | 38173 | 34 |

*Note.* AFM, affected family member (i.e., primary victim); Respondent, person alleged to have perpetrated family violence; np, values are not published to protect confidentiality; General pop., Victorian general population.

**Figure A1**. Mental illness emergency department presentation rates per 1,000 people (from 28 February 2017 to 31 December 2018) for family violence affected family members, respondents, and the general population, by age group and sex. *Legend.* AFM, affected family member (i.e., primary victim); Respondent, person alleged to have perpetrated family violence. The data for this graph are included in Table A4.


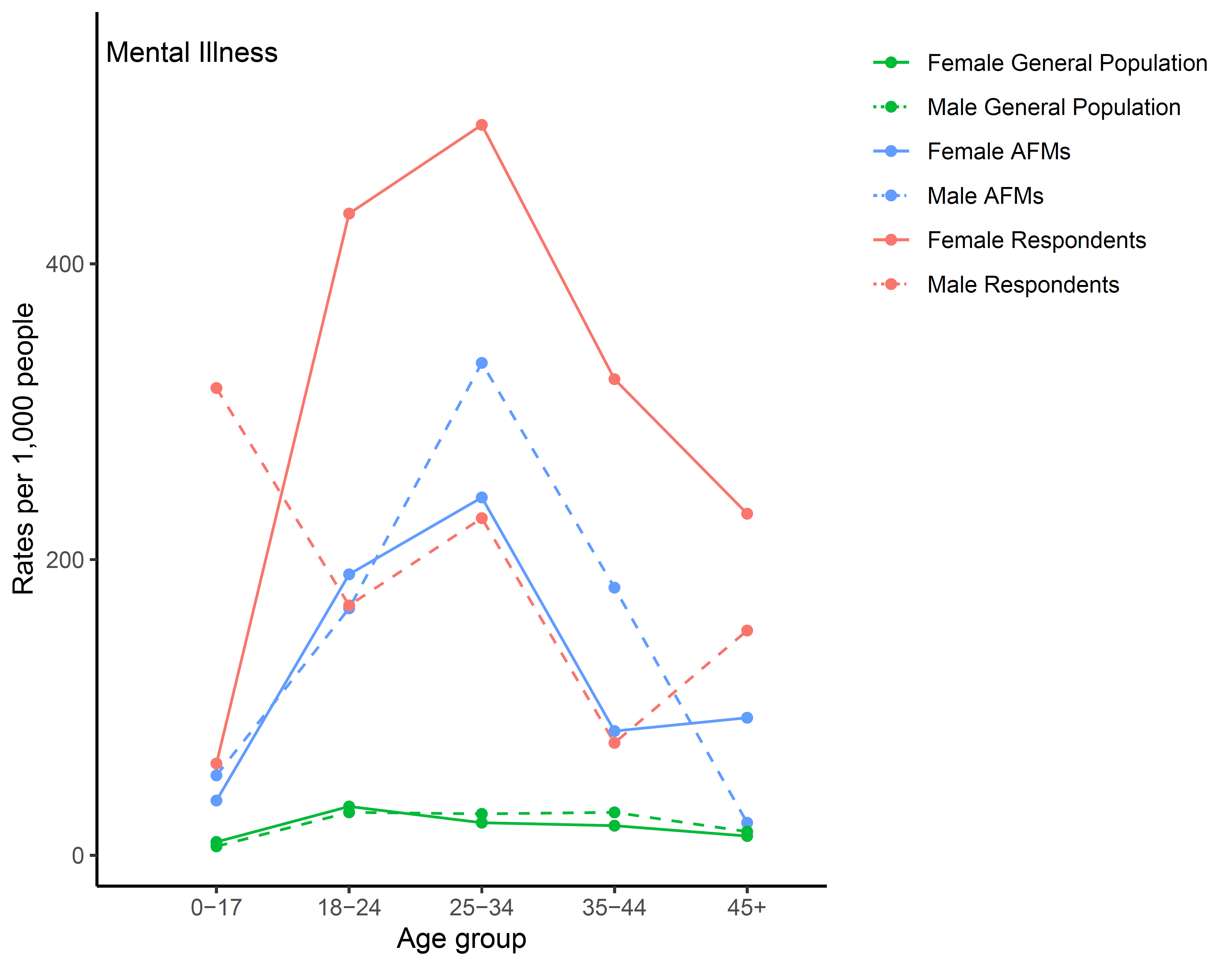


**Figure A2**. Self-harm emergency department presentation rates per 1,000 people (from 28 February 2017 to 31 December 2018) for family violence affected family members, respondents, and the general population, by age group and sex. *Legend.* AFM, affected family member (i.e., primary victim); Respondent, person alleged to have perpetrated family violence. The data for this graph are included in Table A4.

**Figure A3**. Other mental health emergency department presentation rates per 1,000 people (from 28 February 2017 to 31 December 2018) for family violence affected family members, respondents, and the general population, by age group and sex. *Legend.* AFM, affected family member (i.e., primary victim); Respondent, person alleged to have perpetrated family violence. The data for this graph are included in Table A4.

Table A5 and A6 report the descriptive data and bivariate analyses pertaining to the associations between family violence risk factors and future mental health emergency department (ED) presentations (any), for affected family members (i.e., primary victims) and respondents (i.e., persons alleged to have perpetrated family violence), respectively. Among both groups, characteristics associated with significantly increased odds of a future mental presentation were depression or mental health issues, suicidal ideation or attempts, alcohol and drug use concerns, a history of mental health ED presentations, and prior police-reported family violence as an affected family member or respondent. Controlling behaviours toward the affected family member by the respondent was also associated with higher odds of a mental health ED presentations for affected family members only, whereas two additional significant correlates were identified for respondents: unemployment and no known recent relationship separation.

**Table A5.** Unadjusted ORs and 95% CIs representing the associations between family violence risk and vulnerability factors recorded by police at the index incident report and future mental health ED presentations (any; from the index family violence report to December 31 2018), among family violence affected family members.

| **Risk or Vulnerability Factor** | **Overall Sample**  N = 1,520  *n* (%) | **No Future Mental Health ED Presentation**  N = 1,345  *n* (%) | **Future Mental Health ED Presentation**  N = 175  *n* (%) | OR | 95% CI | *p*-value |
| --- | --- | --- | --- | --- | --- | --- |
| Depression or mental health issues |  |  |  | 3.41 | 2.32, 5.01 | <0.001 |
| Not Present/Unknown | 1,351 (88.9%) | 1,221 (90.8%) | 130 (74.3%) |  |  |  |
| Present | 169 (11.1%) | 124 (9.2%) | 45 (25.7%) |  |  |  |
| Suicidal ideation or attempts |  |  |  | 8.18 | 3.61, 18.5 | <0.001 |
| Not Present/Unknown | 1,496 (98.4%) | 1,333 (99.1%) | 163 (93.1%) |  |  |  |
| Present | 24 (1.6%) | 12 (0.9%) | 12 (6.9%) |  |  |  |
| Isolation |  |  |  | 0.92 | 0.49, 1.70 | 0.8 |
| Not Present/Unknown | 1,408 (92.6%) | 1,245 (92.6%) | 163 (93.1%) |  |  |  |
| Present | 112 (7.4%) | 100 (7.4%) | 12 (6.9%) |  |  |  |
| Alcohol use |  |  |  | 2.02 | 1.33, 3.06 | <0.001 |
| Not Present/Unknown | 1,348 (88.7%) | 1,206 (89.7%) | 142 (81.1%) |  |  |  |
| Present | 172 (11.3%) | 139 (10.3%) | 33 (18.9%) |  |  |  |
| Drug use |  |  |  | 4.63 | 3.16, 6.79 | <0.001 |
| Not Present/Unknown | 1,363 (89.7%) | 1,238 (92.0%) | 125 (71.4%) |  |  |  |
| Present | 157 (10.3%) | 107 (8.0%) | 50 (28.6%) |  |  |  |
| Recent separation |  |  |  | 1.27 | 0.80, 2.01 | 0.3 |
| Not Present/Unknown | 1,346 (88.6%) | 1,195 (88.8%) | 151 (86.3%) |  |  |  |
| Present | 174 (11.4%) | 150 (11.2%) | 24 (13.7%) |  |  |  |
| Escalation |  |  |  | 1.15 | 0.77, 1.72 | 0.5 |
| Not Present/Unknown | 1,261 (83.0%) | 1,119 (83.2%) | 142 (81.1%) |  |  |  |
| Present | 259 (17.0%) | 226 (16.8%) | 33 (18.9%) |  |  |  |
| Financial difficulties |  |  |  | 1.41 | 0.84, 2.36 | 0.2 |
| Not Present/Unknown | 1,394 (91.7%) | 1,238 (92.0%) | 156 (89.1%) |  |  |  |
| Present | 126 (8.3%) | 107 (8.0%) | 19 (10.9%) |  |  |  |
| Harm or threat to harm the AFM |  |  |  | 1.07 | 0.71, 1.62 | 0.7 |
| Not Present/Unknown | 1,264 (83.2%) | 1,120 (83.3%) | 144 (82.3%) |  |  |  |
| Present | 256 (16.8%) | 225 (16.7%) | 31 (17.7%) |  |  |  |
| Sexual assault of AFM |  |  |  | 1.10 | 0.38, 3.17 | 0.9 |
| Not Present/Unknown | 1,488 (97.9%) | np | np |  |  |  |
| Present | 32 (2.1%) | np | np |  |  |  |
| Controlling behaviours by respondent |  |  |  | 1.48 | 1.01, 2.18 | 0.045 |
| Not Present/Unknown | 1,263 (83.1%) | 1,127 (83.8%) | 136 (77.7%) |  |  |  |
| Present | 257 (16.9%) | 218 (16.2%) | 39 (22.3%) |  |  |  |
| Pregnancy or recent birth |  |  |  | 1.22 | 0.57, 2.61 | 0.6 |
| Not Present/Unknown | 1,461 (96.1%) | 1,294 (96.2%) | 167 (95.4%) |  |  |  |
| Present | 59 (3.9%) | 51 (3.8%) | 8 (4.6%) |  |  |  |
| Prior mental health ED presentation |  |  |  | 7.45 | 5.32, 10.4 | <0.001 |
| Not Present | 1,155 (76.0%) | 1,091 (81.1%) | 64 (36.6%) |  |  |  |
| Present | 365 (24.0%) | 254 (18.9%) | 111 (63.4%) |  |  |  |
| Prior police reported FV as AFM |  |  |  | 1.67 | 1.21, 2.32 | 0.002 |
| Not Present | 715 (47.0%) | 652 (48.5%) | 63 (36.0%) |  |  |  |
| Present | 805 (53.0%) | 693 (51.5%) | 112 (64.0%) |  |  |  |
| Prior police reported FV as respondent |  |  |  | 3.30 | 2.39, 4.55 | <0.001 |
| Not Present | 1,062 (69.9%) | 983 (73.1%) | 79 (45.1%) |  |  |  |
| Present | 458 (30.1%) | 362 (26.9%) | 96 (54.9%) |  |  |  |

*Note.* FV, family violence; ED, emergency department; AFM, affected family member (i.e., primary victim); Respondent, person alleged to have perpetrated family violence; OR, odds ratio; CI, confidence interval; np, values are not published to protect confidentiality.

**Table A6.** Unadjusted ORs and 95% CIs representing the associations between family violence risk and vulnerability factors recorded by police at the index incident report and future mental health ED presentations (any; from the index family violence report to December 31 2018), among family violence respondents.

| **Risk or Vulnerability Factor** | **Overall Sample**  N = 1,470  *n* (%) | **No Future Mental Health ED Presentation**  N = 1,218  *n* (%) | **Future Mental Health ED Presentation**  N = 252  *n* (%) | OR | 95% CI | *p*-value |
| --- | --- | --- | --- | --- | --- | --- |
| Depression or mental health issues |  |  |  | 3.05 | 2.25, 4.12 | <0.001 |
| Not Present/Unknown | 1,196 (81.4%) | 1,033 (84.8%) | 163 (64.7%) |  |  |  |
| Present | 274 (18.6%) | 185 (15.2%) | 89 (35.3%) |  |  |  |
| Suicidal ideation or attempts |  |  |  | 4.68 | 2.68, 8.18 | <0.001 |
| Not Present/Unknown | 1,417 (96.4%) | 1,190 (97.7%) | 227 (90.1%) |  |  |  |
| Present | 53 (3.6%) | 28 (2.3%) | 25 (9.9%) |  |  |  |
| Unemployment |  |  |  | 2.03 | 1.39, 2.95 | <0.001 |
| Not Present/Unknown | 1,307 (88.9%) | 1,100 (90.3%) | 207 (82.1%) |  |  |  |
| Present | 163 (11.1%) | 118 (9.7%) | 45 (17.9%) |  |  |  |
| Alcohol use |  |  |  | 2.08 | 1.55, 2.81 | <0.001 |
| Not Present/Unknown | 1,146 (78.0%) | 979 (80.4%) | 167 (66.3%) |  |  |  |
| Present | 324 (22.0%) | 239 (19.6%) | 85 (33.7%) |  |  |  |
| Drug use |  |  |  | 2.26 | 1.70, 3.00 | <0.001 |
| Not Present/Unknown | 1,086 (73.9%) | 936 (76.8%) | 150 (59.5%) |  |  |  |
| Present | 384 (26.1%) | 282 (23.2%) | 102 (40.5%) |  |  |  |
| Recent separation |  |  |  | 0.57 | 0.35, 0.93 | 0.026 |
| Not Present/Unknown | 1,298 (88.3%) | 1,065 (87.4%) | 233 (92.5%) |  |  |  |
| Present | 172 (11.7%) | 153 (12.6%) | 19 (7.5%) |  |  |  |
| Escalation |  |  |  | 1.18 | 0.83, 1.68 | 0.4 |
| Not Present/Unknown | 1,225 (83.3%) | 1,020 (83.7%) | 205 (81.3%) |  |  |  |
| Present | 245 (16.7%) | 198 (16.3%) | 47 (18.7%) |  |  |  |
| Financial difficulties |  |  |  | 0.71 | 0.41, 1.23 | 0.2 |
| Not Present/Unknown | 1,348 (91.7%) | 1,112 (91.3%) | 236 (93.7%) |  |  |  |
| Present | 122 (8.3%) | 106 (8.7%) | 16 (6.3%) |  |  |  |
| Harm or threat to harm the AFM |  |  |  | 1.11 | 0.77, 1.58 | 0.6 |
| Not Present/Unknown | 1,225 (83.3%) | 1,018 (83.6%) | 207 (82.1%) |  |  |  |
| Present | 245 (16.7%) | 200 (16.4%) | 45 (17.9%) |  |  |  |
| Sexual assault of AFM |  |  |  | 0.74 | 0.26, 2.14 | 0.6 |
| Not Present/Unknown | 1,440 (98.0%) | np | np |  |  |  |
| Present | 30 (2.0%) | np | np |  |  |  |
| Controlling behaviours by respondent |  |  |  | 0.74 | 0.50, 1.09 | 0.13 |
| Not Present/Unknown | 1,224 (83.3%) | 1,006 (82.6%) | 218 (86.5%) |  |  |  |
| Present | 246 (16.7%) | 212 (17.4%) | 34 (13.5%) |  |  |  |
| Pregnancy or recent birth |  |  |  | 0.88 | 0.43, 1.82 | 0.7 |
| Not Present/Unknown | 1,412 (96.1%) | 1,169 (96.0%) | 243 (96.4%) |  |  |  |
| Present | 58 (3.9%) | 49 (4.0%) | 9 (3.6%) |  |  |  |
| Prior mental health ED presentation |  |  |  | 8.19 | 6.07, 11.0 | <0.001 |
| Not Present | 1,040 (70.7%) | 961 (78.9%) | 79 (31.3%) |  |  |  |
| Present | 430 (29.3%) | 257 (21.1%) | 173 (68.7%) |  |  |  |
| Hx of violent behaviour by respondent |  |  |  | 1.12 | 0.74, 1.67 | 0.6 |
| Not Present/Unknown | 1,292 (87.9%) | 1,073 (88.1%) | 219 (86.9%) |  |  |  |
| Present | 178 (12.1%) | 145 (11.9%) | 33 (13.1%) |  |  |  |
| Prior police reported FV as AFM |  |  |  | 1.95 | 1.48, 2.57 | <0.001 |
| Not Present | 942 (64.1%) | 814 (66.8%) | 128 (50.8%) |  |  |  |
| Present | 528 (35.9%) | 404 (33.2%) | 124 (49.2%) |  |  |  |
| Prior police reported FV as respondent |  |  |  | 1.73 | 1.28, 2.33 | <0.001 |
| Not Present | 563 (38.3%) | 492 (40.4%) | 71 (28.2%) |  |  |  |
| Present | 907 (61.7%) | 726 (59.6%) | 181 (71.8%) |  |  |  |

*Note.* FV, family violence; AFM, affected family member (i.e., primary victim); Respondent, person alleged to have perpetrated family violence; ED, emergency department; hx, history; OR, odds ratio; CI, confidence interval; np, values are not published to protect confidentiality.
